# Supplementary material for: ProteinShader: illustrative rendering of macromolecules
Source: BMC Struct Biol. 2009 Mar 30;9:19. doi: 10.1186/1472-6807-9-19 (PMC2672931; doi:10.1186/1472-6807-9-19)
Supplement: Additional file 1 — ProteinShader program without source code. This compressed file contains the complete ProteinShader program including associated libraries, but no source code. A README.txt file gives an overview of the ProteinShader distribution, and the index.html file in the help subdirectory has directions on getting started with the program as well as a set of tutorials. [file 1472-6807-9-19-S1.zip › ProteinShader-beta-0_9_4-binary/help/api/org/proteinshader/structure/visitor/enums/class-use/RegionMode.html]

Uses of Class org.proteinshader.structure.visitor.enums.RegionMode (ProteinShader API)


|  |  |  |
| --- | --- | --- |
| **Overview** \| **Package** \| **Class** \| **Use** \| **Tree** \| **Deprecated** \| **Index** \| **Help** | |  |
| PREV   NEXT | **FRAMES**    **NO FRAMES**     **All Classes** |  |


---


## **Uses of Class org.proteinshader.structure.visitor.enums.RegionMode**

| Packages that use RegionMode | |
| --- | --- |
| **org.proteinshader.structure.visitor** | Holds Visitor classes that know how to traverse the hierarchy of objects held by class Structure. |
| **org.proteinshader.structure.visitor.enums** | Holds enums needed by the Visitor classes. |

Uses of RegionMode in org.proteinshader.structure.visitor

| Methods in org.proteinshader.structure.visitor with parameters of type RegionMode | |
| --- | --- |
| `void` | `Visitor.setMode(RegionMode regionMode)`             Sets the traversal mode with regard to Regions: NO\_REGIONS, LOOPS, HELICES, BETA\_STRANDS, or ALL\_REGIONS. |

Uses of RegionMode in org.proteinshader.structure.visitor.enums

| Methods in org.proteinshader.structure.visitor.enums that return RegionMode | |
| --- | --- |
| `static RegionMode` | `RegionMode.valueOf(String name)`             Returns the enum constant of this type with the specified name. |
| `static RegionMode[]` | `RegionMode.values()`             Returns an array containing the constants of this enum type, in the order they're declared. |

---


|  |  |  |
| --- | --- | --- |
| **Overview** \| **Package** \| **Class** \| **Use** \| **Tree** \| **Deprecated** \| **Index** \| **Help** | |  |
| PREV   NEXT | **FRAMES**    **NO FRAMES**     **All Classes** |  |


---

# *Copyright © 2007-2008*
